# F249. FAMILY BURDEN IN THE US RAISE-ETP PROGRAM: TREATMENT EFFECTS AND PREDICTORS

**DOI:** 10.1093/schbul/sby017.780

**Published:** 2018-04-01

**Authors:** Shirley Glynn, Susan Gingerich, Piper Meyer-Kalos, Kim Mueser, Alec Chan-Golston, Catherine Sugar, Nina Schooler, Robert Rosenheck, John Kane

**Affiliations:** 1 UCLA; 2 Independent Consultant; 3 The University of Minnesota; 4 Boston University; 5 SUNY Downstate Medical Center; 6 Yale University; 7 Northwell Health

## Abstract

**Background:**

The Recovery After an Initial Schizophrenia Episode Early Treatment Program (RAISE-ETP) is a US NIMH-funded 34 site cluster randomized controlled trial evaluating the benefits of participation in a multicomponent intervention for first episode psychosis (FEP). Previously, participation in the RAISE-ETP comprehensive specialty care (CSC) program, entitled NAVIGATE, was reported to yield significant participant clinical and functional improvements, compared to customary care (Kane et al, 2016). NAVIGATE included tailored medication, individual resiliency training, family education, and supported education and employment. Family burden has been identified as a key factor in FEP, with high levels of distress often found in relatives. Here, we look at the presence and predictors of family burden in relatives in the RAISE-ETP sample over the two years of study participation.

**Methods:**

A total of 404 individuals between ages 15 and 40 were enrolled. DSM-IV diagnoses of non-affective psychosis were included. All participants had experienced only one episode of psychosis, had been prescribed less than 6 months of lifetime psychotic medication, spoke English, and provided informed consent. Participants were offered a minimum of two years of CSC or customary care. At baseline, participants provided demographic and clinical history information; they were administered the Heinrichs-Carpenter Quality of Life Scale (QOL) and the Positive and Negative Symptom Scale (PANSS) regularly throughout the study. Each participant was asked to nominate a family member for administration of the Burden Assessment Scale (BAS) throughout the study. The BAS yields a total score, as well as subscales assessing disrupted activities, personal distress, guilt, time perspective, and worry.

**Results:**

Fifty-seven percent of the participants nominated a relative who was assessed with the BAS. Interestingly, the only statistically significant independent predictors of baseline family burden were relatives’ reports of their loved ones’ dependence and lack of help with chores; no consumer demographic, PANSS, or QOL variables were identified. BAS total scores improved significantly in both conditions, but significantly more in NAVIGATE. Consumer report of better family relationship quality on the QOL was associated with significantly less family burden on the BAS over time, but neither PANSS positive, negative or symptom total, total QOL, nor participation in specific CSC psychosocial components mediated the observed BAS total burden reductions. With regard to the BAS burden components, there was a main effect of improvement over time on family disrupted activities, guilt, time perspective, and worry, with disrupted activities, personal distress, and guilt all evidencing a time by group interaction favoring greater reductions in NAVIGATE.

**Discussion:**

As anticipated, family burden is widely evidenced in the relatives of US FEP consumers who are new to treatment. This burden does not appear to reflect unique consumer characteristics. There appears to be a reduction in family burden during the loved one’s FEP treatment, with that reduction enhanced when the consumer is participating in a more intensive CSC program. Interestingly, while many potential intervening variables were tested as mediators of the CSC impact on family burden, none were identified. The overall pattern of results suggests that it maybe the very fact of a loved one being enrolled in a treatment program, especially if it is a comprehensive FEP intervention, rather than engagement in specific program components or consumer improvements, that are associated with reductions in family burden over the first year of treatment.

